# Chromosomally and Plasmid-Located *mcr* in Salmonella from Animals and Food Products in China

**DOI:** 10.1128/spectrum.02773-22

**Published:** 2022-11-21

**Authors:** Cai-Yue Mei, Yue Jiang, Qin-Chun Ma, Meng-Jun Lu, Han Wu, Zhen-Yu Wang, Xinan Jiao, Jing Wang

**Affiliations:** a Jiangsu Key Laboratory of Zoonosis/Jiangsu Co-Innovation Center for Prevention and Control of Important Animal Infectious Diseases and Zoonoses, Yangzhou University, Yangzhou, China; b Key Laboratory of Prevention and Control of Biological Hazard Factors (Animal Origin) for Agrifood Safety and Quality, Ministry of Agriculture of China, Yangzhou University, Yangzhou, China; USDA-ARS

**Keywords:** *Salmonella*, *mcr-1*, *mcr-4.3*, plasmid, chromosome

## Abstract

This study aimed to investigate the prevalence and genomic characteristics of the colistin resistance gene *mcr* in Salmonella enterica in China. In total, 445 S. enterica isolates from animals and food products were screened through PCR and sequencing for the presence of *mcr*. The *mcr* genes were detected in nine Salmonella strains (2.02%), with complete *mcr-1* in S. enterica serovar Indiana (*n* = 1) and an *S.* Typhimurium monophasic variant (*S.* 4,[5],12:i:-; *n* = 1), *mcr-4.3* in S. enterica serovar London (*n* = 1), and an incomplete *mcr-1* in *S*. Indiana (*n* = 6). They exhibited MIC values of 0.25 to 8 mg/L to colistin and showed resistance to multiple antimicrobial agents. Whole-genome sequencing was performed on *mcr*-positive Salmonella strains using Illumina HiSeq or PacBio single-molecule real-time sequencing. The complete *mcr-1* gene was located on conjugative IncN1-IncHI2 plasmid and IncX4 plasmid, respectively, with high similarity to other *mcr-1*-bearing plasmids belonging to the same incompatibility type. Together with an additional 13 antimicrobial resistance genes, the incomplete *mcr-1* was embedded in an 81,442-bp multiresistance region on the chromosome in *S*. Indiana YZ20MCS6. The Δ*mcr-1-pap2* segment and a set of tellurite resistance determinants (*terYXWZABCDEF*) in six *S*. Indiana strains were similar to other IncHI2 plasmid backbones. The *mcr-4.3* gene was located on an untyped plasmid pYULZMPS10. Although low prevalence of *mcr* was observed in Salmonella, continuous surveillance of this gene in Salmonella is required. Plasmids play an important role in *mcr* transmission, and *mcr-1*, although incomplete, can be captured by chromosomes with the help of mobile elements.

**IMPORTANCE** Colistin is a last-resort antibiotic for severe infections caused by multidrug-resistant (MDR) Gram-negative pathogens. Colistin resistance genes *mcr*, particularly *mcr-1*, have been found in *Enterobacteriaceae* around the world, mainly in Escherichia coli and Salmonella. Salmonella enterica is a major foodborne pathogen, with MDR Salmonella being considered a “Serious Threat Level pathogen” by the Centers for Disease Control and Prevention. Therefore, the prevalence of *mcr* in Salmonella strains must be monitored. In this study, a low *mcr* prevalence (2.02%) was observed in Salmonella strains from animals and food products, with plasmid-borne *mcr-1* in S. enterica serovar Indiana and an *S.* Typhimurium monophasic variant (*S.* 4,[5],12:i:-) and chromosomally located *mcr-1* in *S.* Indiana. The *mcr-4.3* gene was first identified in S. enterica serovar London associated with an untyped plasmid. Although this study reports a low *mcr* prevalence in Salmonella, the transmission ability of *mcr*-positive Salmonella strains to humans via the food chain is a public health concern.

## INTRODUCTION

Globally, antibiotic-resistant bacteria are a great public health concern, and their emergence is commonly associated with inappropriate use of antibiotics in hospitals and farms. The rapid increase of multidrug-resistant Gram-negative infections gave rise to the restoration of polymyxins, particularly colistin, in therapeutic regimens (1). However, the identification and spread of the plasmid-borne colistin resistance gene *mcr-1* can reduce the clinical efficacy of colistin (1, 2). Until now, 10 known *mcr* homologs (*mcr-1* to *mcr-10*) have been identified globally in multiple species from various sources (3).

Salmonella enterica is one of the most prevalent foodborne pathogens both in humans and animals, particularly nontyphoidal Salmonella, which give rise to an estimated 93.8 million diarrheal illnesses with 155,000 deaths annually, making it a focused point of surveillance by the World Health Organization (4, 5). Notably, among Gram-negative species, the prevalence of *mcr* in S. enterica is second only to that in Escherichia coli, and Salmonella enterica serovar Typhimurium is the most prevalent serotype carrying *mcr* globally (1, 6, 7). Different *mcr* variants, including *mcr-1*, *mcr-2*, *mcr-3*, *mcr-4*, *mcr-5*, and *mcr-9*, have been detected in Salmonella isolated from humans, animals, food, and the environment, with *mcr-1* being the most frequent variant (6). *mcr* is spreading among Salmonella globally, possibly because this gene can rapidly transfer horizontally between bacteria, which is frequently mediated by different plasmids (6, 7).

The reduction of colistin resistance and prevalence of *mcr-1* was observed after the use of colistin as a feed additive was discontinued in China on April 30, 2017 (8). Considering the public health threat posed by *mcr*-positive Salmonella, we investigated the prevalence of *mcr* in Salmonella from food-producing animals and retail meat and assessed the genomic characteristics of *mcr* in this study.

## RESULTS AND DISCUSSION

### Characterization of *mcr-*positive Salmonella isolates.

We detected *mcr* genes in 445 previously obtained S. enterica strains from food-producing animals and retail meat products from 2019 to 2021 in China (9). Among them, seven S. enterica serovar Indiana strains from chicken meat and one *S.* Typhimurium monophasic variant (*S.* 4,[5],12:i:-) from pigs were positive for *mcr-1*, and one S. enterica serovar London strain from pork was positive for *mcr-4* (Table S1 in the supplemental material). The detailed information of these *mcr*-positive isolates is presented in Table 1. No other *mcr* gene was detected. According to the multilocus sequencing typing (MLST) analysis based on whole-genome sequencing, all *S*. Indiana strains belonged to sequence type (ST) 17, and *S.* 4,[5],12:i:- and *S*. London isolates were classified as ST34 and ST155, respectively.

**TABLE 1:** Characteristics of *mcr*-carrying Salmonella isolates in this study

Strain^*a*^	Source	Serotype	ST	*mcr*	Other resistance genes	Colistin MIC (mg/L)	Other resistance patterns^*b*^	Mutations		Location of *mcr*
								*gyrA*	*parC*	
YZ20MCS5	Chicken meat, 2020	Indiana	17	Δ*mcr-1*	*bla*_CTX-M-55_, *bla*_OXA-1_, *armA*, *aph*(3′)*-Ia*, *aph*(4)*-Ia*, *aac*(3)*-IVa*, *aadA1*, *aadA2*, *aadA5*, *strAB*, *tet*(A), *cmlA1*, *catB3*, *floR*, *aac*(6′)*-Ib-cr*, *oqxAB*, *fosA3*, *sul1*, *sul2*, *sul3*, *dfrA17*, *mph*(A), *mph*(E), *msr*(E), *arr-3*	0.5	AMP/CFZ/CTX/GEN/AMI/TET/ CHL/FFC/NAL/CIP/FOS/SXT	S83F D87N	S80R	Chromosome
YZ20MCS6	Chicken meat, 2020	Indiana	17	Δ*mcr-1*	*bla*_CTX-M-55_, *bla*_OXA-1_, *armA*, *aac*(6′)*-Iaa*, *aph*(3′)*-Ia*, *aph*(4)*-Ia*, *aac*(3)*-IVa*, *aadA1*, *aadA2*, *aadA5*, *strAB*, *tet*(A), *cmlA1*, *catB3*, *floR*, *aac*(6′)*-Ib-cr*, *oqxAB*, *fosA3*, *arr-3*, *sul1*, *sul2*, *sul3*, *dfrA17*, *mph*(A), *mph*(E), *msr*(E), *arr-3*	0.5	AMP/CFZ/CTX/GEN/AMI/TET/ CHL/FFC/NAL/CIP/FOS/SXT	S83F D87N	S80R	Chromosome
YZ20MCS7	Chicken meat, 2020	Indiana	17	Δ*mcr-1*	*bla*_CTX-M-55_, *bla*_OXA-1_, *aph*(3′)*-Ia*, *aph*(4)*-Ia*, *aac*(3)*-IVa*, *aac*(6′)*-Iaa*, *armA*, *aadA1*, *aadA2*, *aadA5*, *strAB*, *tet*(A), *cmlA1*, *floR*, *aac*(6′)*Ib-cr*, *catB3*, *oqxAB*, *fosA3*, *sul1*, *sul2*, *sul3*, *dfrA17*, *mph*(A), *mph*(E), *msr*(E), *arr-3*	0.5	AMP/CFZ/CTX/GEN/AMI/TET/ CHL/FFC/NAL/CIP/FOS/SXT	S83F D87N	S80R	Chromosome
YZ20MCS8	Chicken meat, 2020	Indiana	17	Δ*mcr-1*	*bla*_CTX-M-55_, *bla*_OXA-1_, *aph*(3′)*-Ia*, *aph*(4)*-Ia*, *aac*(3)*-IVa*, *aac*(6′)*-Iaa*, *armA*, *aadA1*, *aadA2*, *aadA5*, *strAB*, *tet*(A), *cmlA1*, *floR*, *aac*(6′)*Ib-cr*, *catB3*, *oqxAB*, *fosA3*, *sul1*, *sul2*, *sul3*, *dfrA17*, *mph*(A), *mph*(E), *msr*(E), *arr-3*	0.5	AMP/CFZ/CTX/GEN/AMI/TET/ CHL/FFC/NAL/CIP/FOS/SXT	S83F D87N	S80R	Chromosome
YZ20MCS14	Chicken meat, 2020	Indiana	17	*mcr-1*	*bla*_CTX-M-55_, *bla*_TEM-1_, *bla*_OXA-1_, *aph*(4)*-Ia*, *aac*(3)*-IVa*, *aadA5*, *aadA22*, *strAB*, *rmtB*, *tet*(A), *floR*, *aac*(6′)*Ib-cr*, *catB3*, *oqxAB*, *fosA3*, *sul1*, *sul2*, *dfrA17*, *mph*(A), *lnu*(F), *arr-3*	2	AMP/CFZ/CTX/GEN/AMI/STR/ TET/CHL/FFC/NAL/CIP/FOS/SXT	S83F D87N	S80R	IncN1-IncHI2 plasmid
AH20MCS1	Chicken meat, 2020	Indiana	17	Δ*mcr-1*	*bla*_CTX-M-55_, *aph*(3′)*-Ia*, *aph*(4)*-Ia*, *aac*(3)*-IVa*, *aadA1*, *aadA2*, *aadA5*, *strAB*, *tet*(A), *cmlA1*, *floR*, *oqxAB*, s*ul2*, *sul3*, *dfrA17*, *mph*(A)	0.5	AMP/CFZ/CTX/STR/TET/ CHL/FFC/NAL/CIP/SXT	S83F D87N	S80R	Chromosome
AH20MCS2	Chicken meat, 2020	Indiana	17	Δ*mcr-1*	*bla*_CTX-M-55_, *aph*(3′)*-Ia*, *aph*(4)*-Ia*, *aac*(3)*-IVa*, *aadA1*, *aadA2*, *aadA5*, *strAB*, *tet*(A), *cmlA1*, *floR*, *oqxAB*, *sul2*, *sul3*, *dfrA17*, *mph*(A)	0.25	AMP/CFZ/CTX/STR/TET/ CHL/FFC/NAL/CIP/SXT	S83F D87N	S80R	Chromosome
GD19PS1	Pig, 2019	4,[5],12:i:-	34	*mcr-1*	*bla*_TEM-1B_, *bla*_OXA-1_, *aac*(3)*-VIa*, *aac*(6′)*-Iaa*, *aph*(3′)*-Ia*, *aph*(4)*-Ia*, *aadA1*, *aadA2*, *strAB*, *tet*(B), *cmlA1*, *catB3*, *floR*, *aac(6′)Ib-cr*, *oqxAB*, *sul1*, *sul2*, *sul3*, *arr-3*	8	AMP/GEN/STR/TET/CHL/ FFC/NAL/CIP/SXT	D87Y	None	IncX4 plasmid
LZ19MPS10	Pork, 2019	London	155	*mcr-4.3*	*bla*_TEM-135_, *aadA1*, *aadA16*, *strAB*, *tet*(A), *floR*, *aac*(6′)*Ib-cr*, *sul1*, *sul2*, *dfrA27*, *arr-3*, *mph*(A)	0.5	AMP/STR/TET/CHL/FFC/SXT	None	None	Untyped plasmid

a Different locations are indicated as follows: YZ, Yangzhou, Jiangsu Province; AH, Anhui Province; GD, Guangdong Province; LZ, Lanzhou, Gansu Province.

b AMP, ampicillin; CFZ, cefazolin; CTX, cefotaxime; GEN, gentamicin; AMI, amikacin; TET, tetracycline; CHL, chloramphenicol; FFC, florfenicol; NAL, nalidixic acid; CIP, ciprofloxacin; FOS, fosfomycin; SXT, sulfamethoxazole/trimethoprim; STR, streptomycin.

The *mcr-1* gene has been reported in numerous Salmonella serotypes, such as Typhimurium and its monophasic variant, Anatum, Derby, Indiana, London, Rissen, and Newport (6). In the present study, *S*. Indiana ST17 strains were the major vectors for *mcr-1*. *mcr*-*1*-carrying *S*. Indiana ST17 strains were previously identified from patients, poultry slaughterhouses, and various food matrices in China (10–12). In 2017, *mcr-4* was first described in one *S.* 4,[5],12:i:- strain from pigs in Italy (13). Subsequently, *mcr-4* and its variants were detected in *S.* Typhimurium, *S.* 4,[5],12:i:-, and S. enterica serovar Kedougou (7). To our knowledge, this is the first case of *mcr-4* detection in *S.* London.

The *mcr*-positive isolates had MIC values determined for 15 antimicrobial agents, including colistin. Surprisingly, seven of the *mcr*-positive Salmonella isolates were susceptible to colistin, with MIC values of 0.25 to 0.5 mg/L, and the remaining isolates YZ20MCS14 and GD19PS1 exhibited reduced susceptibility (MIC = 2 mg/L) or resistance (MIC = 8 mg/L) to colistin (Table 1). All *mcr*-positive Salmonella isolates showed resistance to ampicillin, tetracycline, chloramphenicol, florfenicol, sulfamethoxazole/trimethoprim, and some other agents (Table 1).

Interestingly, an analysis of *mcr*-positive contigs from the draft genomes of Salmonella isolates sequenced by Illumina revealed that six *S*. Indiana strains had incomplete *mcr-1*, which thus retained susceptibility to colistin. The intact *mcr-1* gene was observed in *S*. Indiana YZ20MCS14 (MIC = 2 mg/L) and colistin-resistant Salmonella 4,[5],12:i:- GD19PS1. The *S*. London isolate LZ19MPS10 carried an *mcr-4* variant, namely, *mcr-4.3*, which was first described in Enterobacter cloacae, with a silent phenotype due to two mutations (V179G and V236F) (14). In addition to *mcr*, all isolates contained multiple antimicrobial resistance genes consistent with their susceptibility profiles, such as *bla*_CTX-M-55_ (cephalosporin resistance), *floR* (florfenicol resistance), and *fosA3* (fosfomycin resistance) (Table 1). In addition, all *S*. Indiana strains had mutations in *gyrA* (S83F and D87N) and *parC* (S80R), and *S.* 4,[5],12:i:- had a single mutation in *gyrA* (D87Y); all mutations were responsible for their resistance to ciprofloxacin.

### Characterization of the *mcr-1*-bearing IncN1-IncHI2 plasmid pYUYZMCS14-1 in the *S*. Indiana strain YZ20MCS14.

**FIG 1:**
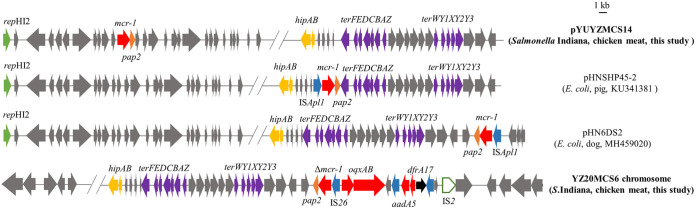
The insertion of *mcr-1* in plasmid pYUYZMCS14-1 and chromosome of YZ20MCS6 in this study and structural comparison with related *mcr-1*-carrying IncHI2 plasmids. The arrows indicate the positions and the transcription directions of the genes. The delta symbol (Δ) indicates a truncated gene or mobile element.

The complete genome sequence of *S*. Indiana YZ20MCS14 was obtained and was composed of one chromosome (4,808,467 bp) and two plasmids (Table S2). The *mcr-1* gene was located on a 229,976-bp plasmid pYUYZMCS14-1, classified as IncHI2/ST3 plasmid with similar organization to other IncHI2 plasmids found in Salmonella (Fig. S1). However, the *mcr-1*-*pap2* structure was inserted close to the IncHI2 replication protein RepHI2, which was different from the previously described *mcr-1*-bearing IncHI2 plasmids (Fig. 1). Additionally, insertion sequence IS*Apl1*, commonly associated with *mcr-1*, was absent in pYUYZMCS14-1, possibly ensuring the stability of *mcr-1* before plasmid-mediated transmission (15).

In addition, pYUYZMCS14-1 contained multiple resistance genes, such as *rmtB*, *fosA3*, *floR*, *tet*(A), *bla*_TEM-1_, and *bla*_OXA-1_ (Fig. S1), which allow for *mcr-1* coselection under other drugs (e.g., florfenicol and tetracycline), thus accelerating *mcr-1* transmission even after colistin was banned as a growth promoter in China. Furthermore, pYUYZMCS14-1 harbored a 3,050-bp IncN1 segment (Δ*repA*-Iterons I-*resP*-ΔIS*26*) containing the replication initiation gene *repA* truncated by IS*26*, 28 tandem 37-bp repeats within the Iterons I region, a CUP6 repeat region, the putative resolvase gene *resP*, and an incomplete copy of IS*26*. A similar insertion of the IncN replication region has been previously described in the *mcr-1*-carrying IncHI2 plasmids, such as pASSD2-MCR1 (*S.* Typhimurium, pig, GenBank accession number KX856065) and pHN6DS2 (E. coli, dog, MH459020) in China (16).

To investigate the transferability of the *mcr-1*-carrying plasmid pYUYZMCS14-1, conjugation experiments were conducted using the streptomycin-resistant E. coli strain C600 as recipients. pYUYZMCS14-1 was successfully transferred from *S*. Indiana YZ20MCS14 to E. coli C600 at a frequency of 5.85 ± 1.09 × 10^−4^ transconjugants/recipient. Interestingly, the transconjugant showed a higher MIC value (4 mg/L) than its original strain YZ20MCS14 (2 mg/L).

### Characterization of the *mcr-1*-bearing IncX4 plasmid pYUGDPS1-2 in the *S*. 4,[5],12:i:- strain GD19PS1.

The complete sequence of *S.* 4,[5],12:i:- GD19PS1 was obtained (Table S2). The *mcr-1* gene was located on a 33,858-bp IncX4 plasmid designated pYUGDPS1-2. This *mcr-1*-carrying plasmid was organized similar to other *mcr-1*-bearing IncX4 plasmids, such as pB1 (LC479452), pPY1 (KX711708), and pFS170G (KX711707) from E. coli, pmcr1_IncX4 (KU761327) from Klebsiella pneumonia, and pN17-0346 (CP031291) from Salmonella (Fig. S2). All of them shared the same IncX4 backbone, including regions for replication, maintenance and stability, and conjugal transfer, but they differed slightly in regions surrounding *mcr-1* (Fig. S2). In pYUGDPS1-2, the *mcr-1*-*pap2* segment was inserted into the plasmid backbone, and IS*Apl1*, which was observed downstream of *mcr-1*-*pap2* in pPY1, was absent in pYUGDPS1-2. One copy of IS*26* was present around *mcr-1* in some IncX4 plasmids, such as pmcr1_IncX4 and pFS170G, but was absent in pYUGDPS1-2 (Fig. S2). As previously described (17), the IncX4 plasmid pYUGDPS1-2 carried only one resistance gene, *mcr-1*, and was successfully transferred to E. coli C600 at a frequency of 2.79 ± 0.59 × 10^−3^ transconjugants/recipient.

### Genetic context of incomplete *mcr-1* in chromosomes of *S*. Indiana strains.

**FIG 2:**
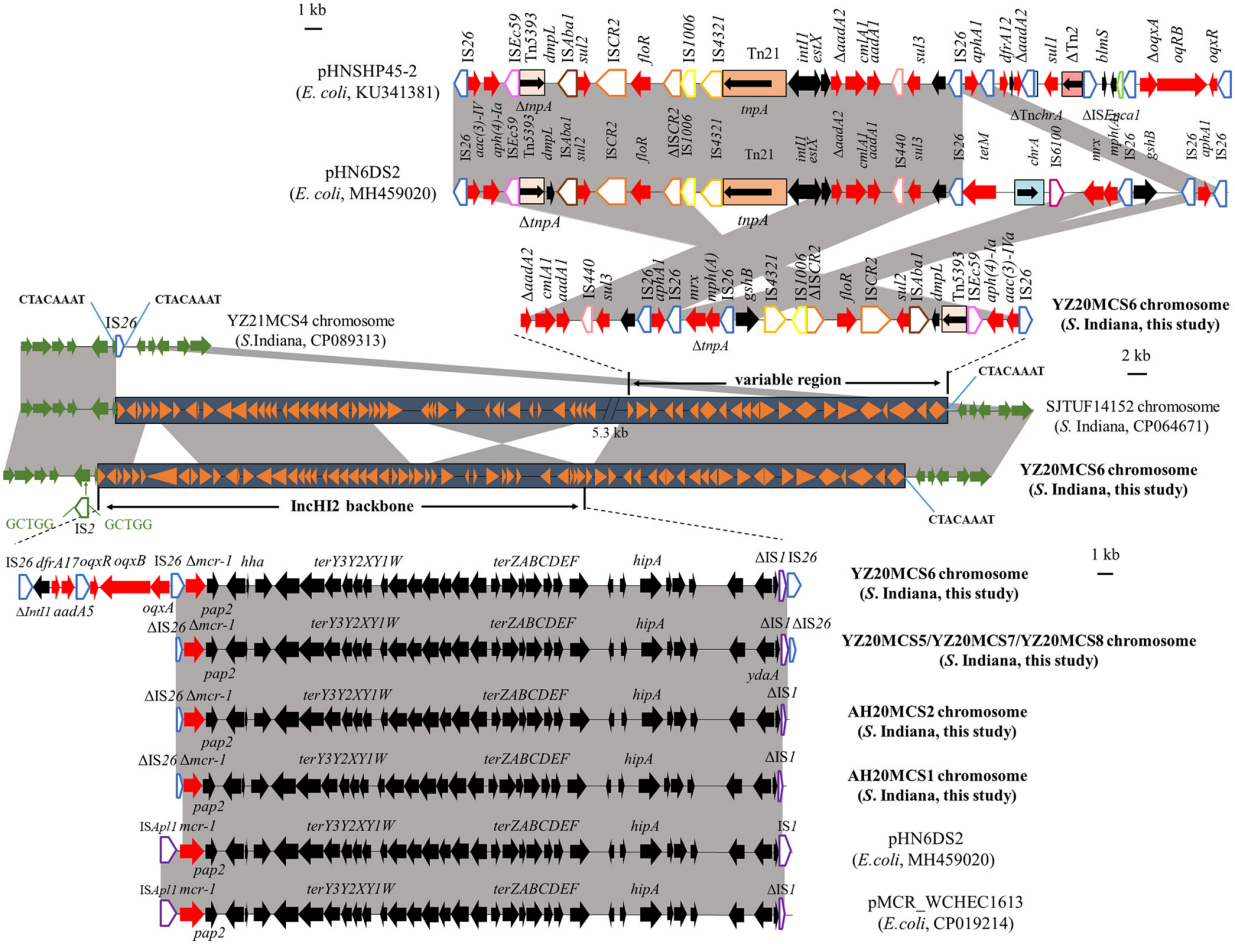
Genetic structures of the multiresistance region in the chromosome of the isolate YZ20MCS6 in this study and comparison with other plasmids or chromosomes. Regions with >99% identity are shaded in gray. Resistance genes are shown in red. Genes on the chromosome of S. Indiana are shown in green. The delta symbol (Δ) indicates a truncated gene or mobile element. ISs are shown as boxes labeled with their name. Labeled vertical arrows with IS box indicate the insertion site of the IS element. Direct repeats are indicated by arrows and sequences.

The complete genome sequence of *S*. Indiana YZ20MCS6, as representative of the *S*. Indiana strain harboring incomplete *mcr-1*, was obtained and was composed of one chromosome (4,990,287 bp) and one 3,373-bp plasmid (Table S2). All antimicrobial resistance genes were located on the chromosome of YZ20MCS6, and an incomplete *mcr-1* (1,311 bp) was embedded in an 81,442-bp multiresistance region (MRR). We further analyzed this MRR and found that *mcr-1* was truncated by IS*26* at the 5′ end (Fig. 2). The Δ*mcr-1-pap2* segment and a set of tellurite resistance determinants (*terYXWZABCDEF*) were similar to other IncHI2 plasmid backbones, such as pHN6DS2 (E. coli, dog, MH459020) and pMCR_WCHEC1613 (E. coli, sewage, CP019214) (Fig. 2). Furthermore, this MRR also contained numerous resistance genes, including *aph*(4)*-Ia*, *aphA1*, *aac*(3)*-IVa*, *aadA1*, Δ*aadA2*, *aadA5*, *cmlA1*, *floR*, *oqxAB*, *sul2*, *sul3*, *mph*(A), and *dfrA17*, and various insertion sequences and transposons, such as IS*26*, IS*1*, IS*4321*, IS*1006*, IS*CR2*, IS*Aba1*, Tn*5393*, and IS*Ec59*. The MRR of YZ20MCS6 was related to those of *mcr-1*-carrying IncHI2 plasmids, such as pHN6DS2 and pHNSHP45-2 (Fig. 2). They differed by rearrangement, insertions, or deletions of multiple segments involving resistance genes, such as *oqxAB*, *aadA2*, *cmlA1*, *aadA1*, *sul3*, *aphA1*, and *tet*(M), and the IncN replication region or other functions, possibly mediated by mobile elements such as IS*26* (Fig. 2). Compared with our previously described *S*. Indiana strain YZ21MCS4 (chicken meat, CP089313) (9) and multiple *S*. Indiana strains, one copy of IS*26* flanked by 8-bp direct repeats (DRs; 5′-CTACAAAT-3′) was inserted into the chromosome at the same right site with identical right lateral DRs without the ~81.4 kb MRR fragment (Fig. 2). A similar insertion of a related MRR was observed in the *S*. Indiana strain SJTUF14152 (chicken meat, CP064671) at the same site (Fig. 2). Thus, we speculate that one copy of IS*26* was first inserted into the chromosome, followed by more acquisition events via IS*26* and/or other mobile elements. Notably, the extensively drug-resistant *S*. Indiana ST17 strain has emerged due to numerous chromosomally located resistance genes (9). More importantly, resistance genes are capable of vertical transfer within this lineage.

We further compared the contigs containing incomplete *mcr-1* in other *S*. Indiana strains. The *mcr-1-*carrying contigs (37,614 to 38,308 bp) in YZ20MCS5, YZ20MCS7, YZ20MCS8, AH20MCS1, and AH20MCS2 were identical to the corresponding region in YZ20MCS6, but a 260-bp shorter *mcr-1* was observed in strain AH20MCS1 (Fig. 2). The IS*26*-interrupted *mcr-1* may explain the colistin-susceptible phenotype in six *S*. Indiana strains. A similar inactivation of *mcr-1* has been previously described to be due to the insertion of mobile elements (e.g., IS*10* and IS*Apl1*) (12, 18). Interestingly, inactivated *mcr-1* due to IS*1294* insertion can be reactivated by losing IS*1294* after colistin selection (19).

### Genetic context of *mcr-4.3* in the plasmid pYULZMPS10 in the *S.* London strain LZ19MPS10.

**FIG 3:**
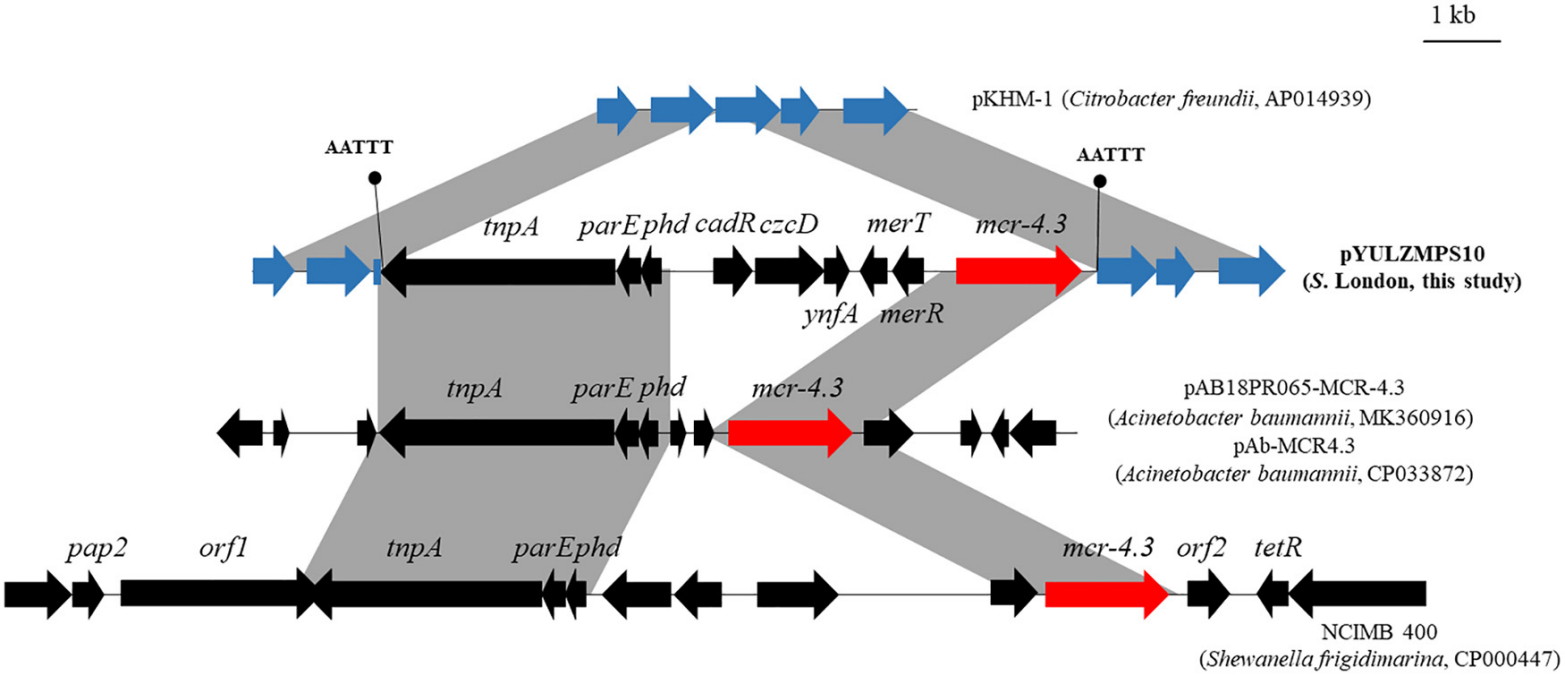
Genetic structure of *mcr-4.3* in plasmid pYULZMPS10 and comparison with other related plasmids or chromosomes. The arrows indicate the positions and the transcription directions of the genes. Regions with >99% identity are shaded in gray. Direct repeats are indicated by arrows and sequences.

The *mcr-4.3* gene was located on the 158,615-bp plasmid designated pYULZMPS10 in *S.* London LZ19MPS10. The pYULZMPS10 backbone showed 99.56% identity to an untyped plasmid pKHM-1 (Citrobacter freundii, AP014939) (Fig. S3). A 9,481-bp region including *mcr-4.3* and additional eight open reading frames was inserted into a hypothetical protein encoding exonuclease belonging to the plasmid backbone, generating 5-bp DRs (5′-AATTT-3′) (Fig. 3). The 3,822-bp segment (*tnpA*-*parE*-*phd*) and 2,191-bp fragment containing *mcr-4.3* showed 99.9% identity to the corresponding region in plasmids pAB18PR065-MCR-4.3 (MK360916) and pAb-MCR4.3 (CP033872) from Acinetobacter baumannii strains and the genome of Shewanella frigidimarina NCIMB 400 (CP000447) (Fig. 3).

The *mcr-4.3* gene was first identified on a ColE10-type plasmid obtained from E. cloacae in 2014 (14). Following this discovery, *mcr-4.3* was also detected on ColE plasmid or untypeable plasmids in A. nosocomialis, A. baumannii, E. coli, Leclercia adecarboxylata, and *E. kobei* (20–22). In this study, *mcr-4.3* was located on an untyped plasmid, indicating that this gene could be captured by different plasmids. Although *mcr-4.3* cannot confer a colistin resistance phenotype, two recovery mutations (G179V and F236V) in *mcr-4.3* can restore the activity of lipid A enzymes and thus confer colistin resistance (23), suggesting the potential of *mcr-4.3* to induce colistin resistance under colistin selection.

### Conclusion.

In summary, the detection rate (2.02%) of *mcr* genes among Salmonella isolates investigated in this study was low, which is consistent with the infrequent prevalence of *mcr* in Salmonella compared with E. coli and the reduction of *mcr* after limited use of colistin in food-producing animals (1, 8). Two strains contained plasmid-borne *mcr-1*, and six *S*. Indiana strains carried chromosomally located *mcr-1*. Additionally, the silenced variant *mcr-4.3* was captured by an untyped plasmid and transferred to *S*. London. Coselection posed by other antibiotics may be responsible for *mcr* transmission.

## MATERIALS AND METHODS

### Detection of *mcr* genes and antimicrobial susceptibility testing.

From July 2019 to April 2021, 1,194 fecal samples from animals (pigs, chickens, and cattle) were collected from farms and slaughterhouses located in six provinces (Anhui, Liaoning, Jiangsu, Guangdong, Shandong, and Xinjiang) of China, and 1,582 food samples (pork, chicken meat, and beef) were collected from farmer’s markets and supermarkets in Anhui, Liaoning, Gansu, Guizhou, Henan, Hubei, Jiangsu, Guangdong, Shandong, and Xinjiang provinces and Shanghai in China. The samples were incubated in buffered peptone water (BPW) broth for 18 to 24 h at 37°C. Then, the enriched BPW suspension (1 mL) was transferred to Rappaport-Vassiliadis R10 broth (10 mL) and incubated for 24 h at 42°C. The positive growth was further streaked on xylose lysine Tergitol 4 (XLT4) agar plates, and the plates were incubated at 37°C for 24 h. One isolate per plate was randomly selected to detect the *stn* gene for Salmonella identification through PCR (24). Serotype identification was performed using commercial diagnostic sera for Salmonella (Ningbo Tianrun Biopharmaceutical, China) based on the White-Kauffmann scheme.

In total, 445 S. enterica strains, including serovars London (*n* = 54), Derby (*n* = 50), Typhimurium (*n* = 46), Rissen (*n* = 46), Kentucky (*n* = 44), Indiana (*n* = 36), Corvallis (*n* = 36), and others (*n* = 133), were previously obtained from food-producing animals (33 pigs, 74 chickens, and 8 cattle) and retail meat products (185 pork, 126 chicken meat, and 19 beef products) (9). The presence of *mcr* genes was detected by PCR and sequencing using primers listed in Table S3 in the supplemental material. The *mcr*-positive Salmonella isolates were tested for their susceptibility to 15 antimicrobial agents by using the broth microdilution method or the agar dilution method (limited to fosfomycin). The results were interpreted according to CLSI Supplement M100, 30th edition (25). The results for florfenicol (>16 mg/L) were interpreted according to the European Committee on Antimicrobial Susceptibility Testing epidemiological cutoff value for S. enterica (www.eucast.org). E. coli ATCC 25922 was used for quality control.

### Conjugation experiments.

To test the transferability of *mcr-1*, conjugation experiments were conducted using colistin-nonsusceptible Salmonella isolates as the donor strains and streptomycin-resistant E. coli C600 as the recipient strain, as previously described (26). Transconjugants were selected on LB agar containing 2 mg/L colistin and 3,000 mg/L streptomycin and confirmed through *mcr* screening and antimicrobial susceptibility tests. The conjugation frequency for *mcr* was calculated as the number of transconjugants per recipient, and experiments were performed in triplicate.

### Whole-genome sequencing and analysis.

Genomic DNA of all *mcr*-positive Salmonella isolates was extracted using the TIAN amp bacteria DNA kit (Tiangen, Beijing, China) following standard protocols. All the *mcr*-positive isolates were sequenced using the Illumina HiSeq platform, and sequence reads were assembled into contigs using SPAdes v.3.8.2. Plasmid contigs were assembled into a complete plasmid sequence by using PCR and Sanger sequencing (limited to pYULZMPS10; Table S4). The serotypes were analyzed by Salmonella *In Silico* Typing Resource (SISTR) (27). The whole genomes of three isolates (*S*. Indiana YZ20MCS6 and YZ20MCS14 and *S*. 4,[5],12:i:- GD19PS1) were further sequenced using PacBio single-molecule real-time sequencing. Raw sequences were introduced into the nonhybrid Hierarchical Genome Assembly Process version 4. Multilocus sequencing typing (MLST), resistance genes, chromosomal mutations, and plasmid replicons were analyzed using the Center for Genomic Epidemiology (CGE) pipelines (http://www.genomicepidemiology.org/). The *mcr*-carrying contigs, plasmids, or chromosomes were analyzed and annotated using RAST (https://rast.nmpdr.org/rast.cgi), ISfinder (https://www-is.biotoul.fr/), and BLAST (https://blast.ncbi.nlm.nih.gov/Blast.cgi).

### Data availability.

The sequence data obtained in this study are deposited at GenBank under the accession number PRJNA847635.
