# Supplementary material for: Chromosomally and Plasmid-Located mcr in Salmonella from Animals and Food Products in China
Source: Microbiol Spectr. 2022 Nov 21;10(6):e02773-22. doi: 10.1128/spectrum.02773-22 (PMC9769515; doi:10.1128/spectrum.02773-22)
Supplement: Supplemental file 1 — Supplemental material. Download spectrum.02773-22-s0001.pdf, PDF file, 0.9 MB [file spectrum.02773-22-s0001.pdf]

Table S1 Distribution of *mcr* in 445 *Salmonella enterica* isolates from animals and food products.

Serovars	Sources (no.)						No. of isolates	No. of isolates with *mcr* (%)
	chicken	pig	cattle	chicken meat	pork	beef		
Agona	14			3			17	
Albany				2			2	
Altona				1			1	
Anatum	1			1	4		6	
Bovismorbificans					1		1	
Braenderup				9	1		10	
Cerro				2			2	
Corvallis	33			3			36	
Derby		12	7		30	1	50	
Enteritidis				22			22	
Give				1	1		2	
Goldcoast					8		8	
Indiana	10			26			36	7 (19.44)
Infantis	1			1	1		3	
Kedougou				1			1	
Kentucky	4	1		38	1		44	
Kottbus				2			2	
Litchfield					6		6	
Livingstone					4		4	
London		4		3	31	16	54	1 (1.85)
Mbandaka	4		1		1		6	
Meleagridis	1						1	
Muenchen	2						2	
Muenster	3						3	
Newport					10		10	
Paratyphi B	1						1	
Putten				2			2	
Rissen		8		2	35	1	46	
Saintpaul				2	7		9	
Senftenberg					1		1	
Thompson				4			4	
Typhimurium		8		1	36	1	46	1 (2.17%)*
Uganda					7		7	

*The *mcr*-positive *S.* Typhimurium was classified as *S.* Typhimurium monophasic variant (*S.* 4,[5],12:i:-) by whole genome sequencing.

Table S2 Complete genome sequences of *mcr*-positive *Salmonella* isolates in this study

	Size (bp)	Resistance genes	plasmid replicon
**YZ20MCS14**			
chromosome	4,808,467	*aac(6')-Iaa*/$bla_{CTX\text{-}M\text{-}55}$	
pYUYZMCS14-1	229,976	$bla_{TEM\text{-}1}$/$bla_{OXA\text{-}1}$ /*aac(3)-IV*/*aph(4)-Ia*/*aadA5*/*aadA22*/*strAB*/ *rmtB*/ *tet*(A)/*floR*/*catB3*/*oqxAB*/*aac(6')-Ib-cr*/*mcr-1*/*fosA3*/*sul1*/*sul2*/*dfrA17*/ *mph*(A)/*Inu*(F)/*arr-3*	IncN1-IncHI2
pYUYZMCS14-2	3,372	none	/
**GD19PS1**			
chromosome	4,963,890	$bla_{TEM\text{-}1B}$/*aac(6')-Iaa*/*strAB*/*tet*(B)/*sul2*	
pYUGDPS1-1	241,147	$bla_{OXA\text{-}1}$/*aac(3)-IV*/*aph(4)-Ia*/*aph(3')-Ia/aadA1*/*aadA2*/*cmlA1*/*floR*/ *catB3*/*oqxAB*/*aac(6')-Ib-cr*/*sul1*/*sul2*/*sul3*/ *arr-3*	IncHI2
pYUGDPS1-2	33,858	*mcr-1*	IncX4
pYUGDPS1-3	3,554	none	/
**YZ20MCS6**			
chromosome	4,990,287	$bla_{OXA\text{-}1}$/$bla_{CTX\text{-}M\text{-}55}$/*aac(6')-Iaa*/*aac(3)-IV*/*aph(4)-Ia*/*aph(3')-Ia*/ *aadA1*/*aadA2*/*aadA5*/*strAB*/*armA*/*tet*(A)/*cmlA1*/*floR*/*catB3*/*oqxAB*/*aac(6')-Ib-cr*/ Δ*mcr-1*/*fosA3*/*sul1*/*sul2*/*sul3*/*dfrA17*/*mph*(A)/ *mph*(E)/*msr*(E)/*arr-3*	
pYUYZMCS6	3,373	none	/

Table S3 Primers used for PCR in this study

Gene	Primer name	Sequence(5' to 3')	Size (bp)	Reference
mcr-1	mcr-1-F	TGCCAATCTACTCGG	536	This study
	mcr-1-R	GTCATCTAAGCCAACG		
mcr-2	mcr-2-F	TGGTACAGCCCCTTTATT	1617	Xavier et al., 2016
	mcr-2-R	GCTTGAGATTGGGTTATGA		
mcr-3	mcr-3-F	TTGGCACTGTATTTTGCATTT	542	Yin et al., 2017
	mcr-3-R	TTAACGAAATTGGCTGGAACA		

mcr-4	mcr-4-F	ATTGGGATAGTCGCCTTTTT	487	Carattoli et al., 2017
	mcr-4-R	TTACAGCCAGAATCATTATCA		
mcr-5	mcr-5-F	ATGCGGTTGTCTGCATTTATC	1644	Borowiak et al., 2017
	mcr-5-R	TCATTGTGGTTGTCCTTTTCTG		
mcr-6	mcr-6-F	AGCTATGTCAATCCCGTGAT	252	Borowiak et al., 2020
	mcr-6-R	ATCACGGGATTGACATAGCTAC		
mcr-7	mcr-7-F	GCCCTTCTTTTCGTTGTT	551	Borowiak et al., 2020
	mcr-7-R	GGTTGGTCTCTTTCTCGT		
mcr-8	mcr-8-F	TCAACAATTCTACAAAGCGTG	856	Borowiak et al., 2020
	mcr-8-R	AATGCTGCGCGAATGAAG		
mcr-9	mcr-9-F	TTCCCTTTGTTCTGGTTG	1011	Borowiak et al., 2020
	mcr-9-R	GCACCTAATAAGTCGGTC		
mcr-10	mcr-10-F	GGACCGACCTATTACCAGCG	366	Lei et al., 2020
	mcr-10-R	GGCATTATGCTGCAGACACG		

References

1. Borowiak M, Baumann B, Fischer J, Thomas K, Deneke C, Hammerl JA, Szabo I, Malorny B. 2020. Development of a novel *mcr-6* to *mcr-9* multiplex PCR and assessment of *mcr-1* to *mcr-9* occurrence in colistin-resistant *Salmonella enterica* isolates from environment, feed, animals and food (2011-2018) in Germany. Front Microbiol 11: 80. https://doi.org/10.3389/fmicb.2020.00080.
2. Borowiak M, Fischer J, Hammerl JA, Hendriksen RS, Szabo I, Malorny B. 2017. Identification of a novel transposon-associated phosphoethanolamine transferase gene, *mcr-5*, conferring colistin resistance in d-tartrate fermenting *Salmonella enterica* subsp. *enterica* serovar Paratyphi B. J Antimicrob Chemother 72(12): 3317-3324. https://doi.org/10.1093/jac/dkx327.
3. Carattoli A, Villa L, Feudi C, Curcio L, Orsini S, Luppi A, Pezzotti G, Magistrali CF. 2017. Novel plasmid-mediated colistin resistance *mcr-4* gene in *Salmonella* and *Escherichia coli*, Italy 2013, Spain and Belgium, 2015 to 2016. Euro Surveill 22(31): 30589. https://doi.org/10.2807/1560-7917.ES.2017.22.31.30589.

4. Lei CW, Zhang Y, Wang YT, Wang HN. 2020. Detection of mobile colistin resistance gene *mcr-10.1* in a conjugative plasmid from *Enterobacter roggenkampii* of chicken origin in China. Antimicrob Agents Chemother 64:e01191-20. https://doi.org/10.1128/AAC.01191-20.
5. Xavier BB, Lammens C, Ruhal R, Kumar-Singh S, Butaye P, Goossens H, Malhotra-Kumar S. 2016. Identification of a novel plasmid-mediated colistin-resistance gene, *mcr-2*, in *Escherichia coli*, Belgium, June 2016. Euro Surveill 21(27). https://doi.org/10.2807/1560-7917.ES.2016.21.27.30280.
6. Yin W, Li H, Shen Y, Liu Z, Wang S, Shen Z, Zhang R, Walsh TR, Shen J, Wang Y. 2017. Novel plasmid-mediated colistin resistance gene *mcr-3* in *Escherichia coli*. mBio 8(3): e00543-17. https://doi.org/10.1128/mBio.01166-17.

Table S4 Primers used to assemble plasmid pYULZMPS10

Primer name	sequence (5'-3')	Size (bp)	Position
1F	CGTCCTTTCGTGAGA	831	contig14
1R	GAACAGGCCAGTTGA		contig27
2F	CGAAGAAGAGGTGGTT	734	contig27
2R	ATGGGTTATTGAAAGGT		contig14

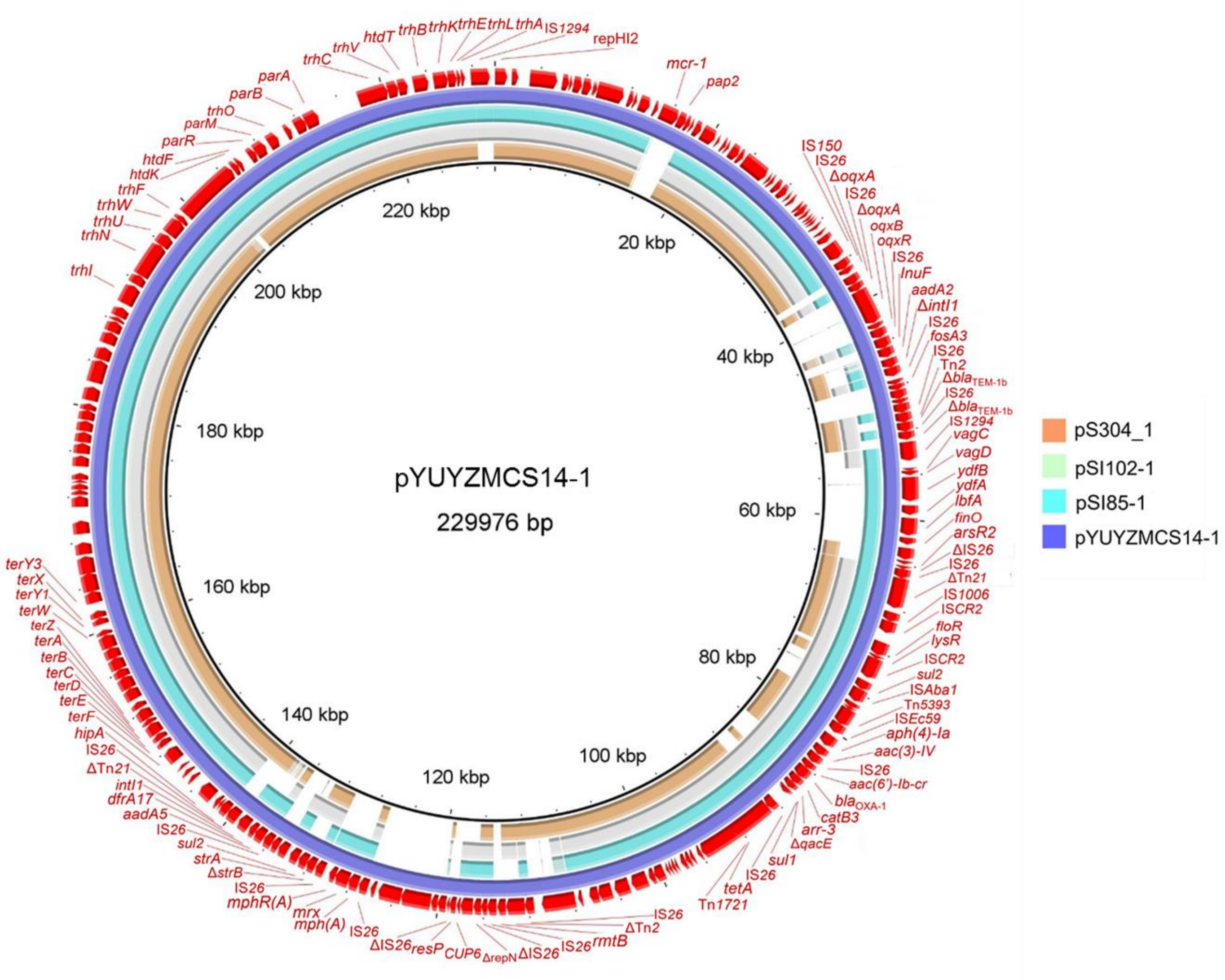

Fig. S1. Sequence comparison of plasmid pYUYZMCS14-1 with plasmids pS304_1 (GenBank accession number CP061127), pSI102-1 (CP050772), and pSI85-1 (CP050780) using BRIG. The reference sequence pYUYZMCS14-1 is indicated in red in the outer circle.

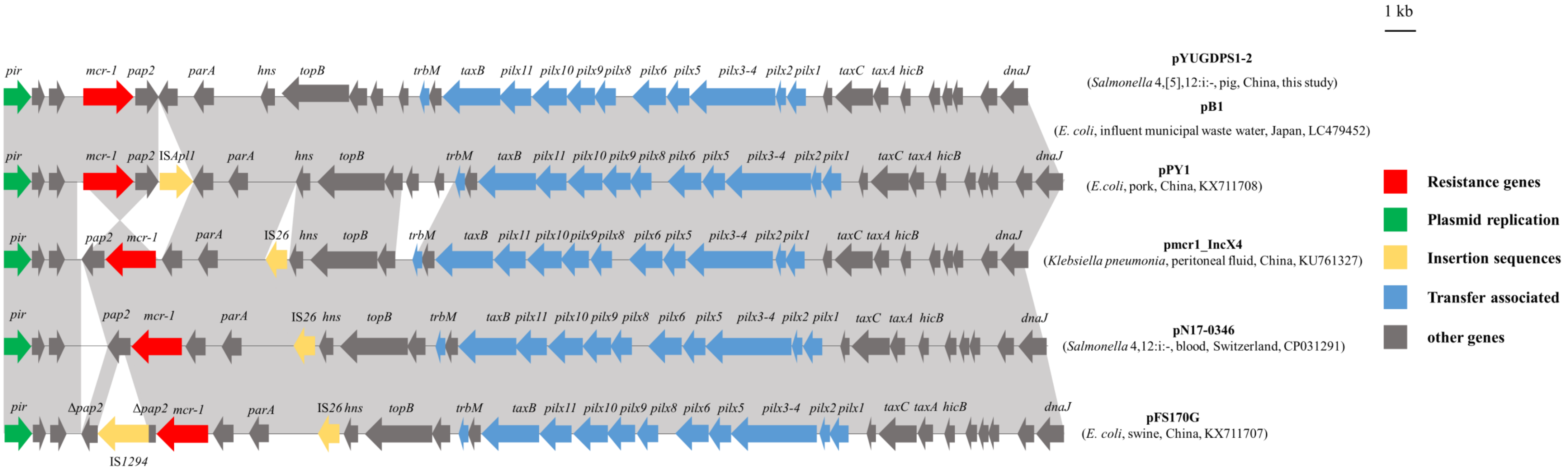

Fig. S2. Linear comparisons of IncX4 plasmid pYUGDPS1-2 with other similar IncX4 *mcr-1*-carrying plasmids. Regions of >99% identity are shaded in grey.

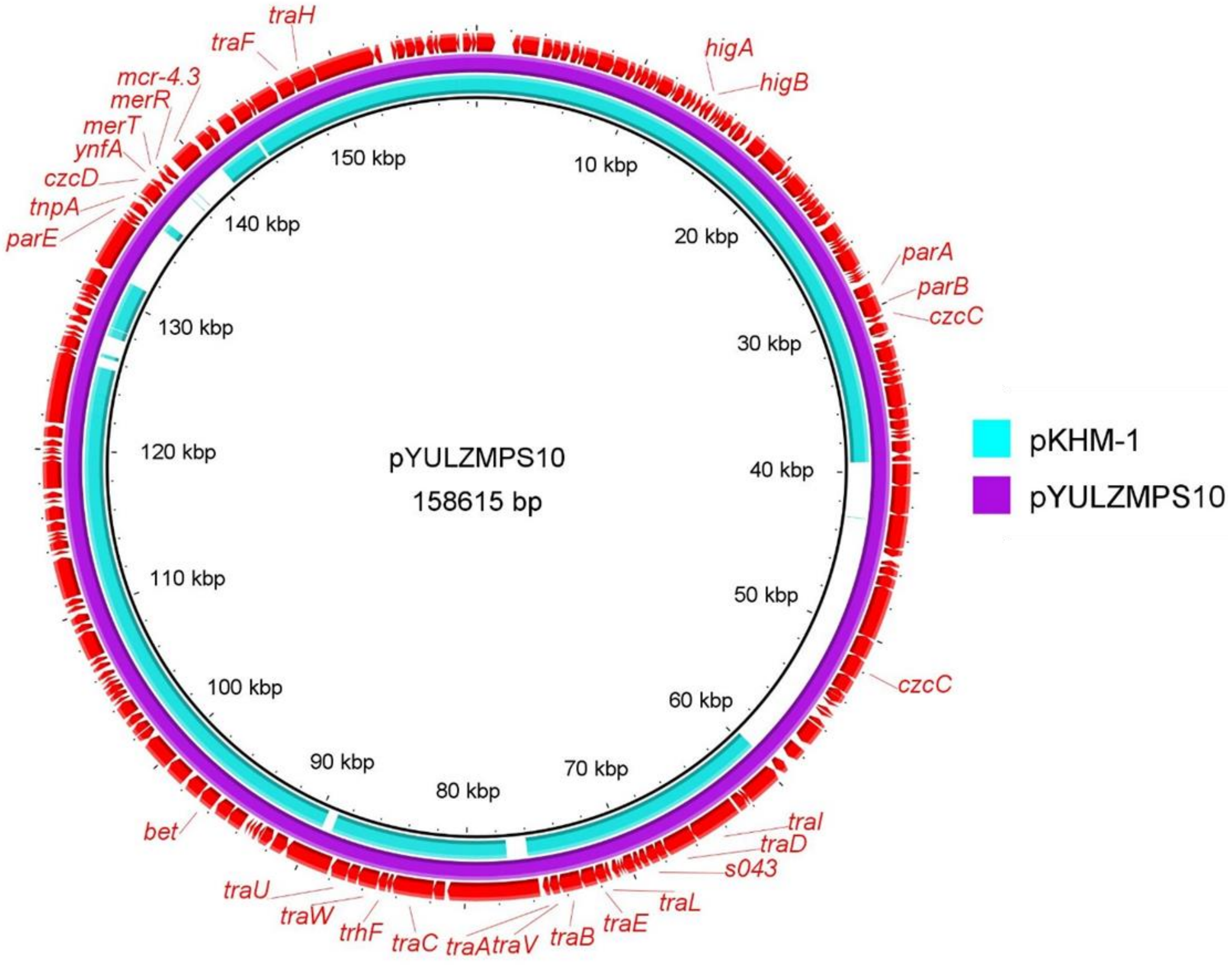

Fig. S3. Sequence comparison of plasmid pYULZMPS10 with plasmid pKHM-1 (AP014939) using BRIG. The reference sequence pYULZMPS10 is indicated in red in the outer circle.
